# ‘It was a ravage!’: lived experiences of epidemic cholera in rural Haiti

**DOI:** 10.1136/bmjgh-2019-001834

**Published:** 2019-11-10

**Authors:** Yodeline Guillaume, Max Raymond, Gregory J Jerome, Ralph Ternier, Louise C Ivers

**Affiliations:** 1 Center for Global Health, Massachusetts General Hospital, Boston, Massachusetts, USA; 2 Zanmi Lasante, Port-au-Prince, Haiti; 3 Department of Global Health and Social Medicine, Harvard Medical School, Boston, Massachusetts, USA

**Keywords:** cholera, Haiti, qualitative study

## Abstract

**Introduction:**

A cholera epidemic began in Haiti over 8 years ago, prompting numerous, largely quantitative research studies. Assessments of local ‘knowledge, attitudes and practices’ relevant for cholera control have relied primarily on cross-sectional surveys. The voices of affected Haitians have rarely been elevated in the scientific literature on the topic.

**Methods:**

We undertook focus groups with stakeholders in the Artibonite region of Haiti in 2011, as part of planning for a public health intervention to control cholera at the height of the epidemic. In this study, we coded and analysed themes from 55 community members in five focus groups, focusing on local experiences of cholera and responses to the prevention messages.

**Results:**

The majority of participants had a personal experience with cholera and described its spread in militaristic terms, as a disease that ‘attacked’ individuals, ‘ravaged’ communities and induced fear. Pre-existing structural deficiencies were identified as increasing the risk of illness and death. Knowledge of public health messages coincided with some improvements in water treatment and handwashing, but not changes in open defecation in their community, and was sometimes associated with self-blame or shame. Most participants cited constrained resources, and a minority listed individual neglect, for inconsistent or unimproved practices.

**Conclusion:**

The experience of epidemic cholera in a rural Haitian community at the beginning of a major outbreak included a high burden and was exacerbated by poverty, which increased risk while hindering practice of known prevention messages. To interrupt cholera transmission, public health education must be paired with investments in structural improvements that expand access to prevention and healthcare services.

**Key questions**

**What is already known?**

- From 2010 to 2018, a cholera epidemic in Haiti resulted in 819 777 suspected cases, disproportionately affecting poor and rural households, and particularly those in regions such as the Artibonite Valley.
- Despite the disease’s impact, there is limited qualitative research on local perceptions and experiences of cholera in Haiti and how these factors might have shaped community responses to the epidemic and related public health messages.

**What are the new findings?**

- We describe local experiences, understandings and responses to cholera in a rural Haitian community before and after the outbreak was identified specifically as ‘cholera’. Community members reported prompt uptake of public health messages and some improvements in water treatment and handwashing but noted that structural deficiencies and poverty remained drivers of cholera transmission and barriers to prevention and treatment efforts.
- Prevention campaigns emphasising individual-level actions caused some community members to feel shame and self-blame, believing that cholera resulted from personal ‘dirtiness’ or ‘neglect’.

**What do the new findings imply?**

- In cholera epidemics, public health education must be matched by appropriate resources to address structural barriers and reduce gaps between water, sanitation and hygiene knowledge and practice.
- Public health campaigns should balance individual-level recommendations with calls for institutional investments and policies that support disease prevention to address shame and self-blame among populations that are often already stigmatised by poverty.

## Introduction

In October 2010, a major epidemic of cholera began in the Central Plateau and Artibonite Valley of Haiti when *Vibrio cholerae* was introduced into an extensive river system by United Nations peacekeepers.^1^ The outbreak rapidly spread to all 10 administrative regions in the country within a month.^2^ In this population, with no prior exposure to the disease^3^ and insufficient safe water and sanitation infrastructure,^4^ the impact was devastating. In the first year of the outbreak alone, the Haitian Ministry of Health reported over half million suspected cases and nearly 7000 deaths. Public health efforts have slowed the epidemic in the subsequent years, reducing incidence from 18.38 in 2010 to 0.30 in 2018, but by then cumulatively 819 777 cholera cases and almost 10 000 deaths had been recorded.^5^

Despite this high burden, little is documented in the scientific literature about the lived experiences of affected Haitians. Most published work has been quantitative and focused on a range of topics including the disease’s origin,^6^ evolution^7^ and the effectiveness of different control strategies.^8^ A smaller strand of the literature has assessed local knowledge, attitudes and practices relevant for prevention, but relying primarily on cross-sectional surveys.^9 10^ Because emphasis has been on informing or evaluating public health interventions, the voices of Haitians who mostly suffered the epidemic’s consequences have not been elevated. Qualitative research on community experiences and responses to the epidemic can provide additional insights into local burden and its contributing factors.

Qualitative studies on perceptions of cholera in areas such as Port-au-Prince, Haiti’s capital, in the western region^11^ and some towns in the northern^12^ and Artibonite^13^ regions of the country revealed great suspicion regarding the outbreak’s origin, widespread fear of contagion and some misconceptions about transmission, prevention and treatment. Some of these studies, however, were conducted less than 2 months into the epidemic when cholera was still relatively new to the population and public health messages were possibly not fully transmitted or understood. Another paper examined the retention of prevention messages in the Artibonite approximately 18 months later, but not lived experiences of cholera.^14^ Other qualitative work on this disease have mainly been conducted outside Haiti.^15–17^

Drawing on focus group data collected in the Artibonite a year after epidemic onset, we aimed to address both cholera burden and understandings from the perspectives and experiences of individuals most directly impacted.

## Methods

### Project area

We undertook focus group discussions (FGDs) in Bocozel, a rural agricultural community in the Artibonite Valley of Haiti, as part of a series of meetings to plan a public health intervention. We intended to explore, within the context of residents’ milieu and realities, the acceptability and feasibility of introducing an oral cholera vaccine as an integrated component of a comprehensive cholera control strategy.^18^

Located in the commune of Saint-Marc, Bocozel had a population of over 30 000 inhabitants,^18^ of which thousands were in close interaction with the cholera-contaminated Artibonite River through canals used for drinking water, bathing, washing and farming purposes.^19^ The area also had a vulnerability to flooding due to its low-lying topography, and limited access to basic water, sanitation, hygiene and healthcare services.^20^ It thus exhibited many suitable conditions for cholera transmission and was among the earliest and hardest hit places, reporting an attack rate between 5.1% and 7.5% in the first 2 years of the epidemic.^2^

### Procedures

Using administrative maps and institutional resources, we identified 42 localities in Bocozel^21^ and recruited a convenience sample of residents with the assistance of community health workers familiar with the area. These workers used a combination of snowball and purposeful sampling strategies to approach potential participants of various occupational backgrounds, both in person and via letters, based on our inclusion criteria for age (18 years or older) and residence.

A project team consisting of native and functionally fluent speakers of the local language developed the initial discussion questions and interview guide in Haitian Creole, focusing on eliciting information around four major themes: (1) lived experiences and interpretations of the first cases and deaths, before the disease was named by health experts and mass media as cholera; (2) current knowledge and practices of public health messages; (3) general knowledge of vaccines and (4) acceptability of a potential oral cholera vaccine campaign. We concluded focus groups when themes reached saturation. Findings related to the first two themes are presented in this paper (see online supplementary appendix A, eg, of the discussion questions).

10.1136/bmjgh-2019-001834.supp1 Supplementary data

Male and female facilitators and note-takers were native Haitian Creole speakers and trained in moderation techniques, note-taking and the management of group dynamics. Generally, a lead moderator (including GJJ) and a note-taker facilitated the discussions in the local language, while a third team member (including RT or LCI) served as observer. All FGDs took place in a private setting. Participants identified themselves by locality rather than name in audio-recordings of the sessions to help protect privacy, and received a transportation stipend to offset the cost of meeting attendance.

### Analysis

Data analysis included an initial debriefing phase and a formal post hoc research analysis.^22^ In the debriefing phase, moderators and note-takers convened to review the notes and identified major themes that emerged from the FGDs. A Haitian note-taker (MR), fluent in English, developed from this process a written summary that documented and translated the highlighted themes, with illustrative quotations, into English. This summary was provided to the observers for review and feedback. During the formal post hoc analysis, a different team member (YG), in consultation with one of the FGD observers (LCI), transcribed and coded the data in Haitian Creole using NVivo software (QSR International, V.11). Coding categories were initially derived from the main discussion questions and then refined inductively based on the emerging themes.^23^ We translated critical statements from the transcripts into English to illustrate key themes and compared our work with the earlier translation done by the bilingual note-taker to ensure accuracy and agreement. We have also kept some Haitian Creole words and phrases in the translated quotations presented in our findings, as examples of the specific terms used by participants. All authors are fluent in both languages.

### Ethics statement

The non-governmental organisation (NGO) Zanmi Lasante conducted the focus groups in support of the Haitian Ministry of Health’s response to the epidemic. We obtained verbal consent from all participants for audio-recording. Except for two major Artibonite towns, Saint-Marc and Gonaïves, all other names referencing individuals or localities in the paper are pseudonyms.

### Patient and public involvement

Patients were not involved in this study.

## Findings

We held five focus groups in November 2011, comprising each 10–12 participants, totalling 55 individuals aged 18 years or older, representing various occupations and more than half of the 42 localities identified in Bocozel, ranging from small rural areas of less than 50 households to larger areas of over 200 households. Two of the focus groups were gender-specific and three mixed gender (see online supplementary appendix B for more details on the characteristics of the groups).

10.1136/bmjgh-2019-001834.supp2 Supplementary data

Results are organised around four key themes that emerged from the data concerning cholera: (1) disease burden in the beginning of the epidemic; (2) knowledge before and during the epidemic; (3) community vulnerabilities and perceived risk and (4) prevention practices and barriers. For additional supporting material on each theme, see online supplementary appendix C.

10.1136/bmjgh-2019-001834.supp3 Supplementary data

### Physical and psychosocial burden of cholera

Over two-thirds of participants either had cholera themselves or knew multiple individuals who had been sick. In their narratives, most framed the outbreak almost as a physical multifront assault, employing common militaristic disease metaphors such as individuals being ‘attacked’, localities ‘ravaged’, and the region overall ‘hit really, really, really hard’ by cholera.

‘Nan [zòn sa], li fè ravaj’ [It ravaged through this area]…I have an 18-year-old son who was ‘atake’ [attacked] by the disease…Even my mother had it too, was attacked. (Female homemaker)

I would barely return from taking someone to [a health center] that I’d have to go again. You take this person and when you come back, it’s either an aunt, a cousin, or someone else. My kid or someone else. I have to go back. Even I, while I was in the fields…it hit me. (Male farmer)

Many deaths were highlighted from these ‘attacks’, especially in villages along the river banks. As participants recounted these experiences, a salient theme of suffering, loss, and helplessness emerged from their descriptions.

There’s a little locality called Vale—in a week, some 5 to 6 people died…We didn’t know what to do. (Male tailor)

People were just dropping. Here in the 5th section [Bocozel]…it was as if the bastion of the disease itself was located here. (Male community representative)

In addition to this physical burden, participants commonly reported people living in fear, sadness and anxiety. This psychological distress was linked with important disruptions in everyday life and social relations, including a rejection of suspected cases and an exodus of fearful residents from affected neighbourhoods.

‘Nou te vrèman pè’ [We were really scared]…There are people who had it, their families still took care of them. But [others]…they were afraid of going near them…People [also] died because when they got it, they would need a taxi driver to transport them. Taxi drivers were afraid of you. (Female farmer)

If [you] didn’t get a grip of yourself, you would have gone crazy. Because no one really stayed put. They were running [because] they saw a disease that was killing people. (Female participant)

Several male farmers further described negative interactions with urban residents who were afraid of coming into contact with people or food from cholera-affected rural areas.

My wife lives in Gonaïves…Before ‘kolera’ [cholera] had reached Gonaïves, [when] I would go to bring her merchandise, some people would run to my side…But now, some would come out, look at me, but wouldn’t come close. ‘Yo vin pè paske zòn kote m soti a kolera ap fè ravaj’ [They’d become afraid because cholera was ravaging through the area that I come from].

Despite fear, a few participants noted the formation of local response committees to coordinate transportation of cases to health facilities, first on their own and later in collaboration with emergency ambulance services.

I sought out five guys who had motorcycles; I bought fuel for them. Whenever someone said they had ‘dyare’ [diarrhoea], they took the person on a motorcycle and quickly brought them to [a health center]…All night long, we were picking up people…When things got worse for us, we had to call ambulances from Saint-Marc. (Male farmer)

### Knowledge of cholera before and during the epidemic

When we asked participants about cholera knowledge prior to the epidemic, almost all indicated unawareness of the disease before its introduction to Haiti.

When the cholera disease was causing ‘ravaj’ [ravage], no one knew what it was. We all heard about people dying…‘Tout 5èm nan nèt, moun ap mouri’ [All over the 5^th^ section of Bocozel, people are dying]…But we didn’t know the disease’s name. (Male community leader)

Three participants claimed to have previously heard of *‘*kolera*’* (the Haitian Creole word for cholera) in reference to diarrhoeal illnesses in Haiti or in news reports of outbreaks in other countries. But two of them acknowledged a misuse of the term for simply ‘any other diarrhoea’.

Lacking previous exposure to epidemic cholera, most remembered an attribution of the first cases and deaths to ‘malefisans’(maleficence): the deliberate use of witchcraft or ‘poud’, a disease-causing powder purportedly lethal following contact.

At the time, in October [2010], we were in the fields and a man named Jean…they came back with him very sick. ‘Se te dyare, vomisman’ [It was diarrhoea, vomiting]…But his family thought that something else was happening with him…like a ‘poud’ used on the streets…a ‘malfesan’ [maleficent person] attacking him. (Female homemaker)

A very small number of participants also reported themselves or others interpreting some early cases as ‘vant pase*’* or ‘usual’ diarrhoea, attributed to gastrointestinal issues such as colic.

As the outbreak rapidly expanded across localities, these initial views were largely abandoned and replaced with news reports or rumours tracing the source of the epidemic to a contamination of the Artibonite River.

The first thing said was, ‘They put something in the river’…No one knew who the ‘they’ were. MINUSTAH [the United Nations peacekeeping troops linked to the introduction of cholera in Haiti] was mentioned later on. (Male community representative)

Such statements, according to several participants, generated scepticism among some residents who reasoned, based on the historical use of the river across many generations, that ‘mikwòb pa touye Ayisyen’ (microbes don’t kill Haitians).

By the time of the focus groups, however, local understandings of cholera generally aligned with public health messages disseminated by the Haitian Ministry of Health and its partners, and which focused on prevention and treatment. Prevention messages, communicated in Haitian Creole and translated here, included content such as: (1) wash your hands with soap and safe water, (2) use treated water, (3) wash fruits and vegetables with treated water, (4) cook all your food thoroughly and (5) throw away stool and vomit in latrines.^24^ Additional content discouraged open defecation and emphasised environmental hygiene or cleanliness.^25^

When queried about their current knowledge, most participants who addressed the cause of cholera associated it with a microbe or unsafe health practices, particularly the ingestion of contaminated food or water and unwashed hands. Two men specifically referred to the pathogen as *V. cholerae*.

It is a germ-causing disease, ‘yon mikwòb’ [a microbe]. (Male farmer)

You can get ‘kolera’ from…untreated water. (Female community leader)

When participants discussed prevention measures, they most frequently listed handwashing and water treatment, followed by proper food handling and an avoidance of open defecation.

We wash our hands, wash them very clean…treat the water so we don’t get it [cholera]. (Female farmer)

A minority further highlighted cleanliness, which paralleled a belief among some participants that cholera ‘loves dirtiness’ or could be transmitted through filthiness.

Don’t put dirty clothes on…so you don’t expose yourself to the ‘mikwòb’ [microbe]. (Male teacher)

Cleanliness is good, because even though there were a lot of people in my compound, only one person had the disease. Because people always took their responsibility, always cleaned their hands and bodies. (Female participant)

Concerning treatment, participants’ responses corresponded with changes in their causal perceptions of cholera. In the early days of the epidemic—when awareness of this specific disease was low—individuals who attributed symptoms to gastrointestinal ailments treated illnesses with home remedies (eg, herbal teas or other local concoctions). If symptoms worsened or led to a death, especially in settings experiencing multiple cases, attempts were made to seek medical care for survivors.

When we saw people dying, you saw people with ‘dyare’ [diarrhoea] and then dying, when you see others ‘atake’ [getting attacked], you bring them to the hospital. (Female participant)

With improved knowledge, the most cited treatment steps included the provision of some form of oral rehydration solution to suspected cases, their transportation to health facilities, or both.

If the person has cholera, just make some ‘sewòm’ [oral rehydration solution] if you haven’t gone to the hospital yet. Take some ‘sewòm’ with you on the way to the hospital. (Male driver)

In addition, the self-medicated use of antibiotics and antidiarrhoeal agents (eg, tetracycline and loperamide) was frequently reported.

### Community vulnerabilities and perceived risk of cholera

Participants described several conditions that placed Bocozel residents at elevated risk for cholera, including the region’s predominantly agriculture-based occupational structure. Many noted farmers’ susceptibility and associated it with their interaction with the contaminated river through irrigation canals and their limited access to potable water and latrines while in the fields.

For some people, it happened in the fields…The person was fine, came from the field and then had the disease. (Female homemaker)

Those who died drank from the water along the road while working in the fields…‘Yo pa jwenn bon dlo trete [tou prè yo] pou yo bwè’ [They can’t find good treated water to drink nearby], so they drink the [untreated] water while working. (Male participant)

Some further pointed to Bocozel’s vulnerability to flooding, which created favourable conditions for cholera transmission.

Every time there’s a storm, it’s on us…[A]s soon as the flooding comes, it’s an ordeal…Water oozes inside the house. (Female homemaker)

[W]hen the river’s in flood…it brings all the feces, all the filthiness near [or] inside our yards, and leaves it there when the water recedes. (Male carpenter)

Others stressed how poor road conditions and limited access to health facilities, which were at times worsened by flooding, contributed to delayed care and preventable deaths, especially with the arrival of cholera in October 2010 coinciding with the rainy season.

It was a bit difficult at the time because of frequent rains. And the roads to get to the upper 5^th^ section are not good at all. So, taking someone to [a health center] was very hard. (Male community representative)

We don’t currently have a health center in the lower 5^th^ section…[And] when it [cholera] first arrived…if someone didn’t see a doctor within 4 hours, he could die. ‘Se sak fè te gen anpil moun ki te mouri’ [That’s why a lot of people died]. (Male community leader)

In this environment, survival from cholera was considered dependent on one’s access to ‘means’ and ‘opportunities’ (eg, family resources, social assistance) that could mediate the exacerbating effects of structural deficiencies. For those lacking such means, death was the expected outcome.

[W]e have…nothing to survive on. If you’re already struggling and this disease is ravaging you and you can’t find anyone to provide some assistance, what’s going to happen to you? Aren’t you going to die? But if there was someone to provide assistance, death wouldn’t come near you. You wouldn’t die prematurely. (Female homemaker)

Several participants used this indirect language of ‘struggle’ or ‘lack of means’ to allude to poverty, or employed words such as ‘someone’ or ‘they’ to refer to those in authority or with the perceived power to address its impact. A few simply observed that communities were abandoned both before and during the epidemic, making no references to unspecified culpable entities.

If the disease had started in the Artibonite, in the 5^th^ section…no one would have come near us, as we were already abandoned as it was. (Male farmer)

‘Zòn lakay mwen manke jwenn sèvis’ [My home area doesn’t receive enough services]…The river water flows down to the locality and people still use it. (Female homemaker)

This sense of abandonment was echoed in evaluation of the early institutional response to cholera, which was deemed too late to prevent the first wave of deaths.

When they came, people were not [dying] anymore. Everyone who were dying had already died. (Male community representative)

Where aid was provided, many claimed that it was insufficient, inequitably distributed, based on friendship or political ties, and coming at first mainly from individuals who were campaigning for political office.

They gave one bar of soap for handwashing, but you have as many as 4 to 5 children in the house. (Female participant)

If you were not in step with [the candidates], you couldn’t receive it…‘Yo fè politik avè l’ [They played politics with it]…They protected us so we’d still be alive to go vote for them. (Male taxi driver)

A minority nevertheless complimented some members of the health, water and sanitation sectors for their assistance.

[One NGO] did a good job. There are people who didn’t have toilets in their compound; they built toilets for them. (Female participant)

### Cholera prevention practices and barriers

Participants highlighted some improvements in health behaviours since epidemic onset. They reported that individuals were more likely to wash their hands and treat their water for drinking and bathing. Some also mentioned making efforts to purchase pretreated water or treatment products, using their own resources, either when communities did not receive such supplies during the emergency response or after free distributions had ended. Their reasons for adopting and maintaining these practices included the high impact of cholera or the risk of illness, which a minority, particularly men, blamed on a collective ‘negligence’ of hygiene measures before the epidemic or persisting individual neglect.

We didn’t usually use Aquatabs [a brand of water purification tablets]. Now, we do. (Female participant)

Till now I always do it [handwashing]. Because it’s something that we should have been doing even before ‘kolera’…It’s our lack of—it’s our ‘neglijans’ [negligence] that allowed [this]. Because cholera had occurred in other places and didn’t inflict all this damage. (Male teacher)

In spite of the cited improvements, some women indicated that poor access to needed resources contributed to inconsistent use of treated water or treatment products.

They use Aquatabs or Clorox [bleach] when they have them. When they don’t, they use the river water.

Our locality is far from [the center of Bocozel], and to buy a gallon of treated water, one would have to go to Saint-Marc. It’s very difficult to come back with it. Even when they do, they don’t use it as [much as] they should. [And] they can’t find Aquatabs in the area.

With respect to sanitation, participants generally reported seeing little to no changes in open defecation practices. They most frequently listed a lack of latrines for the continued behaviour, and financial constraint as the main barrier to their construction.

In my locality, ‘se nan rak preske moun yo fè bezwen yo’ [it’s virtually in the bushes that people defecate]. Even though I work as a health agent telling them of the danger in it… they cannot take actions…Only the few people with some means have a little pit in their yards. You may see those with more means build a latrine with concrete blocks, but others cannot use it. (Male farmer)

Given these obstacles, many wished for some type of governmental or humanitarian assistance to improve prevention practices.

If [this NGO] made a visit, [it]’d see the necessity for building more latrines in this area, because it needs it. (Male carpenter)

‘Leta’ [The state] could take all the necessary measures to provide potable water. (Male teacher)

Despite acknowledging a need for assistance, three women agreed that the government would never be able to subsidise universal coverage and individuals themselves must ultimately take responsibility for sanitation.

There are people who dug pits themselves…and use it to defecate…Sometimes, people use the state as an excuse. But, really, you must help yourselves. By the time to cover the entire country of Haiti—what kind of state would be able to? (Female homemaker)

Although men did not express this view themselves, a few saw an individualistic attitude among latrine owners who were unwilling to share facilities. These owners seemed to convey to non-owners that they should ‘naje pou soti’ (swim their way out) of their circumstances, and not expect assistance from others.

## Discussion

This paper raises the voices of Haitians directly affected by cholera and is a testimony of those most burdened in the first year of the epidemic. Findings showed an array of factors intersecting to create a disastrous experience of the disease in Bocozel, which, in the words of participants, ravaged localities and induced great fear, particularly in the early days of the outbreak. Similar findings have been reported in qualitative studies done in rural and urban communities in both the western and northern parts of the country,^11 12^ where pervasive fear of contamination and cholera had contributed to disruptions in daily life and social interactions.

Pre-existing structural deficiencies, combined with a community vulnerability to flooding, were highlighted for exacerbating cholera impact. More specifically, limited access to treated water, latrines and healthcare facilities—along with the need and challenges of travelling to the main town of Saint-Marc for prevention supplies and urgent medical care—were linked with increased risk of illnesses, suffering and deaths. These views are supported by previous research in Haiti indicating a higher cholera burden among poor and rural households, compared with their more well-off and urban counterparts.^26^

Differences in household outcomes possibly resulted from differential access to water, sanitation, hygiene and healthcare services by socioeconomic and geographical location. Rural Haitians have been shown to be less likely to own latrines and more likely to engage in open defecation,^27^ two factors that augment risk for cholera. They also have lesser access to public and private water services^28 29^ and high-quality healthcare,^30^ all likely contributors to their higher burden. These rural–urban disparities, which are themselves a reflection of large social inequalities in Haiti, helped structure the distribution of cholera risk and outcomes in Bocozel.

Participants were generally aware of the effects of material deprivation but avoided using the term ‘poverty’ when discussing their circumstances, perhaps due to embarrassment associated with this admission.^31^ They instead referred to insufficient resources that exposed residents, especially agricultural workers, to avoidable harm and suffering while restricting access to the tools and services for prevention and, among cases, for prompt treatment. Around the time of the focus groups, nearly 70% of Haitian rural households lived in chronic poverty, confirming their limited means for responding to adverse events.^29^ The lived experience of cholera in Bocozel illustrates the structural violence of this poverty, which had shaped the area’s pre-existing vulnerabilities and made its inhabitants ill equipped to deal with the disease’s arrival and ensuing epidemic.

Despite and within these constraints, the very ‘first responders’ to the outbreak were residents themselves, followed by political candidates with ties to affected localities who provided water and other supplies to affected communities. In addition to the distribution of such emergency products, state institutions and NGOs who responded to the outbreak also reported chlorinating community water supplies, rehabilitating distribution networks and water treatment centres,^4^ expanding health services for prevention and treatment,^24^ and some offered psychosocial support to individuals and communities in order to address the stigma associated with cholera illness and help people mourn their losses.^32^ While many of these interventions were appreciated and sometimes commended, participants considered them too late to prevent many potentially avoidable deaths.

Participants’ evaluation of early response activities and related grievances about being abandoned, underserved or aided in some instances only for political votes hinted at a historical lack of institutional responsiveness to community needs dating prior to the epidemic. Their conclusions further expressed a sense of marginalisation, symptomatic of rural Haitians’ long-standing exclusion from meaningful political participation and provision of services, including those relevant for disease prevention.^33^ Other commentaries suggest that the institutional response to cholera might have been delayed by debates over the origins of the disease and competing public health strategies aimed at controlling the epidemic.^1^

In the context of extreme poverty and unfamiliarity with the disease, cholera was initially attributed to maleficence, or the deliberate use of witchcraft to cause illness. Other early accounts emphasising the introduction of a new pathogen into the river also fit into the ‘maleficence’ narrative, insofar as they suggested the possible, even deliberate, involvement of an external entity or substance in the onset of an epidemic that harmed the population. Similar perceptions about the origins of cholera have been documented in other communities in Haiti,^11 12^ although use of the term ‘maleficence’ did not appear in other studies. The initial attribution of cholera illnesses to witchcraft seemed, however, to have been short lived in Bocozel and replaced with a biomedical and biosocial understanding of the disease. Further, the foreign origin of cholera was only stressed by a minority.

Findings instead indicated a successful and potentially rapid alignment of local knowledge with the disseminated public health messages, and some self-reported improvements in water treatment and handwashing, but not sanitation practices. A survey of poor urban dwellers less than 2 months into the epidemic provides some further evidence of an early awareness of prevention and treatment methods.^9^ Additional research in Haiti also corroborates our findings on the transmission of health messages in the rural Artibonite, with some associated positive changes in water treatment practices.^14 34^ But similar to these and other studies conducted outside Haiti,^35 36^ despite adequate knowledge, resource constraints were cited in the FGDs for sanitation deficiencies and inconsistent water treatment and handwashing.

Since the onset of the epidemic, there have been many initiatives by the government and partnering organisations to increase institutional investments and capacity in the water and sanitation sector and decentralise the provision of services. For example, as part of its strategy to expand access to potable water, the national water agency has promoted household water treatment and safe storage programme, particularly in the rural communities lacking improved water sources. It also reported training and deploying local technicians in rural areas, starting in 2012, to help monitor water quality at distribution points and support related health promotion activities.^4 37^ Assessments of water use and treatment in the Artibonite, within the context of such efforts, have shown an increase in access to improved drinking water sources from 43% of households in 2012 to 59% in 2016.^37^ However, 43% of improved sources tested positive for *Escherichia coli* and inconsistent availability of treatment products persisted. A 2017 national inventory of drinking water infrastructure further showed marked disparities among communities in the Artibonite, with access rates varying from 7% to 74%.^38^ Access to improved sanitation systems is also notably low in this region, with only 18% of the population reporting an on-site, non-shared facility.^26^ Together, these findings reaffirm a need for continued investments in water and sanitation infrastructure to help reduce the transmission of waterborne diseases.

Among a minority of the FGD participants, an awareness of the structural barriers to positive health behaviours coexisted with a perception that poor practices resulted from neglect of prevention messages. This contributed to collective self-blame for the high cholera burden in Haiti or the blaming of cases for their own illnesses, especially when sickness was attributed to filthiness. Previous research suggests that cholera may become linked with filthiness when mass prevention campaigns emphasise the faecal–oral route of transmission.^15 17^ This association in turn can create shame and further marginalisation of vulnerable populations if infection is thought to occur only among those who are filthy or careless.^11^ Cholera cases could subsequently become doubly victimised: first by the disease, and then by the consequences of prevention campaigns intending to use disgust as an agent of behavioural change.

Similar findings have been reported in connection to the use of militaristic disease metaphors to frame epidemics and mass campaigns.^15 39^ Because campaign messages often focus on individual-level actions and tacitly assign the responsibility for ‘fighting’ diseases to target populations, sickness may induce shame and guilt among sufferers if perceived as a personal failure to prevent ‘attacks’ against oneself and communities.^39^ These particular feelings, however, did not emerge among participants from their militaristic descriptions of cholera, perhaps because this framing was neither done at a conscious level nor, to our knowledge, integrated into education materials.

Prevention campaigns in Haiti nonetheless individualised cholera incidence by both placing the burden for controlling the disease on affected persons and overlooking the responsibility of domestic and international institutions^40^—including the United Nations, whose peacekeepers introduced cholera to Haiti—to ensure an adequate response to the epidemic. While individual actions can help protect communities, this emphasis exaggerates the capacity of those impacted, particularly the destitute poor, to act on recommended measures. It also desocialises the context of cholera epidemics and poor health practices by divorcing them from the larger political economic realities of which they emerged.^41^ A more balanced approach to reducing cholera transmission in high-risk communities would pair prevention messages with the supporting tools for individual agency and practice.^42^ This strategy would further include investments in structural improvements and policies that expand access to needed services.

Our findings should be interpreted along with the following limitations. Due to social desirability, positive self-reports of hygiene practices might have been influenced by the normative views expressed within the groups and by the affiliation of moderators with a healthcare organisation. Results are not intended to be generalisable to populations outside the Artibonite, but are used to provide a richer description of the epidemic’s impact on a highly vulnerable rural community beyond that which is possible in a cross-sectional quantitative survey. Despite this, the population studied shares many characteristics with other local communities. Results are also consistent with the previous studies conducted in Haiti, indicating some similarities in people’s perceptions and experiences of cholera.

## Conclusion

The experience of rural Haitians with cholera at the peak of a major epidemic was devastating and shaped by pre-existing structural vulnerabilities, which increased their suffering and restricted their ability to protect themselves against illness. Although aware of prevention measures, they often lacked the means to consistently implement and sustain behavioural changes. In addition, the emphasis of mass prevention campaigns on individual-level actions may have contributed to self-blame and victim blaming among a minority. To interrupt cholera transmission, public health interventions must better address the material needs of the poor in order to strengthen their capacity to translate knowledge into practice. Public health practitioners must also mindfully promote individual behavioural change while advocating for the institutional and infrastructural support relevant for disease prevention.

## References

[1] Frerichs RR Deadly river: cholera and cover-up in post-earthquake Haiti. 1 ed Ithaca, NY: Cornell University Press, 2016.

[2] Barzilay EJ, Schaad N, Magloire R, et al Cholera surveillance during the Haiti epidemic--the first 2 years. N Engl J Med 2013;368:599–609. 10.1056/NEJMoa1204927 23301694

[3] Jenson D, Szabo V, the Duke F, Duke FHI Haiti Humanities Laboratory Student Research Team Cholera in Haiti and other Caribbean regions, 19th century. Emerg Infect Dis 2011;17:2130–5. 10.3201/eid1711.110958 22099117 PMC3310590

[4] Gelting R, Bliss K, Patrick M, et al Water, sanitation and hygiene in Haiti: past, present, and future. Am J Trop Med Hyg 2013;89:665–70. 10.4269/ajtmh.13-0217 24106193 PMC3795096

[5] Ministère de la Santé et de la Population Statistical profile of cholera in the 9th epidemiological week of 2019 [Profil statistique du choléra pour la 9ème semaine épidémiologique de l'année 2019]. Port-au-Prince, Haiti: MSPP, 2019 Available https://mspp.gouv.ht/site/downloads/Profil%20statistique%20Cholera%209SE2019.pdf (accessed 10 July 2019).

[6] Frerichs RR, Keim PS, Barrais R, et al Nepalese origin of cholera epidemic in Haiti. Clin Microbiol Infect 2012;18:E158–E163. 10.1111/j.1469-0691.2012.03841.x 22510219

[7] Katz LS, Petkau A, Beaulaurier J, et al Evolutionary dynamics of Vibrio cholerae O1 following a single-source introduction to Haiti. MBio 2013;4:e00398–13. 10.1128/mBio.00398-13 23820394 PMC3705451

[8] Fung IC-H, Fitter DL, Borse RH, et al Modeling the effect of water, sanitation, and hygiene and oral cholera vaccine implementation in Haiti. Am J Trop Med Hyg 2013;89:633–40. 10.4269/ajtmh.13-0201 24106189 PMC3795092

[9] Beau De Rochars VEM, Tipret J, Patrick M, et al Knowledge, attitudes, and practices related to treatment and prevention of cholera, Haiti, 2010. Emerg Infect Dis 2011;17:2158–61. 10.3201/eid1711.110818 22204033 PMC3310585

[10] Childs L, François J, Choudhury A, Etheart M, Juin S, et al Evaluation of knowledge and practices regarding cholera, water treatment, hygiene, and sanitation before and after an oral cholera vaccination Campaign-Haiti, 2013-2014. Am J Trop Med Hyg 2016;95:1305–13. 10.4269/ajtmh.16-0555 27799642 PMC5154444

[11] Grimaud J, Legagneur F Community beliefs and fears during a cholera outbreak in Haiti. Intervention 2011;9:26–34. 10.1097/WTF.0b013e3283453ef2

[12] Garnett ES Gadyen Dlo “Water Guardian”: a qualitative study of the Influence of cholera on household water treatment practices in Haiti. Atlanta, GA: Rollins School of Public Health of Emory University, 2013.

[13] Gaines J, Gieselman A, Jacobson L, et al Life inside an outbreak: a qualitative assessment of cholera response efforts, Artibonite department, Haiti, 2010. poster 20. 60th annual epidemic intelligence service conference. Atlanta, Georgia, USA. Atlanta: Centers for Disease Control and Prevention, 2011.

[14] Williams HA, Gaines J, Patrick M, et al Perceptions of health communication, water treatment and sanitation in Artibonite department, Haiti, March-April 2012. PLoS One 2015;10:e0142778 10.1371/journal.pone.0142778 26562658 PMC4642927

[15] Nations MK, Monte CM "I'm not dog, no!": cries of resistance against cholera control campaigns. Soc Sci Med 1996;43:1007–24. 10.1016/0277-9536(96)00083-4 8888470

[16] Schaetti C, Khatib AM, Ali SM, et al Social and cultural features of cholera and shigellosis in peri-urban and rural communities of Zanzibar. BMC Infect Dis 2010;10:339 10.1186/1471-2334-10-339 21110853 PMC3009639

[17] Keys HM, Kaiser BN, Foster JW, et al Cholera control and anti-Haitian stigma in the Dominican Republic: from migration policy to lived experience. Anthropol Med 2019;26:123–41. 10.1080/13648470.2017.1368829 29058456

[18] Ivers LC, Teng JE, Lascher J, et al Use of oral cholera vaccine in Haiti: a rural demonstration project. Am J Trop Med Hyg 2013;89:617–24. 10.4269/ajtmh.13-0183 24106187 PMC3795090

[19] Lantagne D, Balakrish Nair G, Lanata CF, et al The cholera outbreak in Haiti: where and how did it begin? Curr Top Microbiol Immunol 2014;379:145–64. 10.1007/82_2013_331 23695726

[20] Haiti Ministry of Agriculture, Natural Resources, and Rural Development General census of agriculture, 2008 community survey [Recensement général de l'agriculture. Enquête communautaire 2008]. Port-au-Prince, Haiti: MARNDR, 2008 http://agriculture.gouv.ht/statistiques_agricoles/EnqueteCommunautaire/documents/DEP05.html (accessed 10 July 2019

[21] Haitian Institute of Statistics and Informatics List of localities and habitations [Liste des localités et habitations]. Port-au-Prince, Haiti: IHSI, 2011.

[22] Nurse J, Woodcock P, Ormsby J Influence of environmental factors on mental health within prisons: focus group study. BMJ 2003;327 10.1136/bmj.327.7413.480 PMC188426 12946970

[23] Fereday J, Muir-Cochrane E Demonstrating rigor using thematic analysis: a hybrid approach of inductive and deductive coding and theme development. Int J Qual Methods 2006;5:80–92. 10.1177/160940690600500107

[24] Tappero JW, Tauxe RV Lessons learned during public health response to cholera epidemic in Haiti and the Dominican Republic. Emerg Infect Dis 2011;17:2087–93. 10.3201/eid1711.110827 22099111 PMC3310587

[25] Pan American Health Organization Sms messages issued by MSPP Haiti on cholera prevention. Washington, D.C: PAHO, 2010 https://www.paho.org/disasters/index.php?option=com_docman&view=download&alias=1382-sms-messages-issued-by-mspp-haiti-on-cholera-prevention&category_slug=docs&Itemid=1179

[26] Cayemittes M, Busangu MF, Bizimana J Earthquake, hunger, and cholera. In: Demographic Health Survey, Haiti 2012 [Enquête Mortalité, Morbidité et Utilisation des Services, Haïti 2012. Calverton, MD: MSPP, IHE and ICF International, 2013: 343–50.

[27] UNICEF Who. progress on sanitation and drinking water – 2015 update and mdg assessment. New York, NY UNICEF; 2015.

[28] Herrera J, Lamaute-Brisson N, Milbin D Changes in living conditions in Haiti between 2007 and 2012: social response to the earthquake [L'évolution des conditions de vie en Haïti entre 2007 et 2012. La réplique sociale du séisme. Port-au-Prince; Paris: IHSI, DIAL, 2014 http://www.ihsi.ht/pdf/ecvmas/analyse/IHSI_DIAL_Rapport%20complet_11072014.pdf

[29] Bank W Poverty and inclusion in Haiti: social gains at timid PACE. Washington, D.C: World Bank Group, 2014 http://documents.worldbank.org/curated/en/643771468257721618/Poverty-and-inclusion-in-Haiti-social-gains-at-timid-pace

[30] Gage AD, Leslie HH, Bitton A, et al Assessing the quality of primary care in Haiti. Bull World Health Organ 2017;95:182–90. 10.2471/BLT.16.179846 28250531 PMC5328114

[31] Koski-Karell V, Farmer PE, Isaac B, et al Haiti's progress in achieving its 10-year plan to eliminate cholera: hidden sickness cannot be cured. Risk Manag Healthc Policy 2016;9:87–100. 10.2147/RMHP.S75919 27307774 PMC4888864

[32] Partners In Health Mourning cholera victims. Boston, MA: PIH, 2011 https://www.pih.org/article/mourning-cholera-victims

[33] Trouillot M-R Haiti’s Nightmare and the Lessons of History. NACLA Report on the Americas 1994;27:46–51. 10.1080/10714839.1994.11724636

[34] Patrick M, Berendes D, Murphy J, et al Access to safe water in rural Artibonite, Haiti 16 months after the onset of the cholera epidemic. Am J Trop Med Hyg 2013;89:647–53. 10.4269/ajtmh.13-0308 24106191 PMC3795094

[35] McMichael C, Robinson P Drivers of sustained hygiene behaviour change: a case study from mid-western Nepal. Soc Sci Med 2016;163:28–36. 10.1016/j.socscimed.2016.06.051 27391250

[36] Sara S, Graham J Ending open defecation in rural Tanzania: which factors facilitate latrine adoption? Int J Environ Res Public Health 2014;11:9854–70. 10.3390/ijerph110909854 25247427 PMC4199054

[37] Centers for Disease Control and Prevention (CDC) Increasing access to improved water and sanitation. Atlanta, GA: CDC, 2018 https://www.cdc.gov/globalhealth/countries/haiti/what/water-and-sanitation.html

[38] Bank W Haiti faced to the challenge of increasing access to drinking water [Haïti face au défi de l’accès l’eau potable. Washington, D.C: World Bank, 2019 https://www.banquemondiale.org/fr/news/feature/2019/03/22/haiti-face-au-defi-de-lacces-a-leau-potable

[39] Sontag S AIDS and its metaphors : Illness as metaphor and AIDS and its metaphors. New York, NY: Doubleday, 1990: 91–124.

[40] Briggs C, Mantini-Briggs C Stories in the time of cholera race and public health in Venezuela. NACLA Report on the Americas 2002;35:43–8. 10.1080/10714839.2002.11722536

[41] Farmer P, Connors M, Fox M, et al Rereading social science : Women, poverty, and AIDS: sex, drugs, and structural violence. farmer P, Connors M, Simmons J. Monroe, ME: Common Courage Press, 2011: 147–205.

[42] Farmer P, Almazor CP, Bahnsen ET, et al Meeting cholera's challenge to Haiti and the world: a joint statement on cholera prevention and care. PLoS Negl Trop Dis 2011;5:e1145 10.1371/journal.pntd.0001145 21655350 PMC3104956

