# Supplementary material for: ‘It was a ravage!’: lived experiences of epidemic cholera in rural Haiti
Source: BMJ Glob Health. 2019 Nov 10;4(6):e001834. doi: 10.1136/bmjgh-2019-001834 (PMC6861088; doi:10.1136/bmjgh-2019-001834)
Supplement: Supplementary data [file bmjgh-2019-001834supp001.pdf]

Supplementary appendix A

Interview guide in Haitian Creole with English translation

General and follow-up questions relating to communities' initial experiences of cholera

- Creole: Kòman nou te viv sitiyasyon kolera a le li te fèk parèt nan zon nan? Lè nou fèk rankontre oswa tande pale de maladi sa, avan nou te tande se te kolera? Kòman nou te viv premye ka yo, lè nou gade moun k ap gen dyare, vomisman, k ap mouri?
- English translation: How did you live or experience the cholera situation when it first appeared in the area? When you first encountered or heard of the disease, before you had heard that it was cholera? How did you experience the first cases, when you saw people having diarrhea, vomiting, dying?

General and follow-up questions relating to cholera knowledge

- Creole: Kisa nou konnen de maladi kolera ? Kijan yon moun ka pran kolera? Ki jan yon moun k ap pwoteje tet li kont kolera?
- English translation: What do you know about the disease of cholera? How can a person get it? How can a person protect him or herself against cholera?
