# Supplementary material for: ‘It was a ravage!’: lived experiences of epidemic cholera in rural Haiti
Source: BMJ Glob Health. 2019 Nov 10;4(6):e001834. doi: 10.1136/bmjgh-2019-001834 (PMC6861088; doi:10.1136/bmjgh-2019-001834)
Supplement: Supplementary data [file bmjgh-2019-001834supp002.pdf]

Supplementary appendix B

Table S1. Characteristics of the focus groups.

Focus group #	Occupational category	Gender
**1**	Farmers, homemakers	Women
**2**	Farmers	Men
**3**	Community representatives	Mix
**4**	School teachers, religious leaders, other community leaders	Mix
**5**	Taxi drivers, small vendors, religious leaders	Mix
