# Supplementary material for: ‘It was a ravage!’: lived experiences of epidemic cholera in rural Haiti
Source: BMJ Glob Health. 2019 Nov 10;4(6):e001834. doi: 10.1136/bmjgh-2019-001834 (PMC6861088; doi:10.1136/bmjgh-2019-001834)
Supplement: Supplementary data [file bmjgh-2019-001834supp003.pdf]

Supplementary appendix C

Table S2. Supplementary data on cholera burden.

**Theme: Physical and psychosocial burden of cholera**	
Sub-theme	Statement
Physical burden	[O]ne of our young men caught the disease…He was having diarrhea, vomiting. Had diarrhea all night…We were waiting for daybreak to go out [for care]. In the morning, around 5, as we were bathing him…he passed away. After that, they told us about Mrs. Pierre who had colic and was vomiting, having diarrhea…We ran out to see her…As we were bathing her, right then and there, she died…In that same evening, I was lying down, I heard one of my children calling me, "Mother, mother! I caught it!"… We didn't know what to do. (Female farmer)
Emotional burden	We lived in sadness. When you heard that a neighbor had it, you started to wonder if you would have it too. When you heard that someone died from *kolera* [cholera], you worried that you would die too. (Female participant)

Disruptions in everyday life and social relations	Even the fishes that people in my locality used to catch…it's our entire livelihood. Everyone abandoned it…You send it to the city, people there say, "He sent me this fish! Is he trying to kill me?" When you get to the city, extend your hand for a handshake, they don't take it. This was very sad. (Male farmer)
	I left my area because 10 people had already died. I then said, "I can't stay." I took my wife and kids and left for Saint-Marc. (Male community leader)

Table S3. Supplementary data on cholera knowledge.

**Theme: Knowledge of cholera before and during the epidemic**	
Sub-theme	Statement
Interpretation of first cases (maleficence)	At first, it was when a boy suddenly died. He died, and everyone was in shock…A second time, again, another person suddenly died. Everyone started to wonder, "Where does this thing come from? Maybe it's a powder attack, a powder that has been brought into the area?" (Male teacher)
Interpretation of first cases ("ordinary" diarrhea)	I had it but didn't know it was cholera at the time…[I]t was just watery diarrhea…all white, with white mucus…I was vomiting…But I thought this was caused by cold weather [because] I work in the fields, in damp conditions. (Female participant)
Interpretation of first cases (river contamination)	I heard one rumor saying that the disease was called *kolera*. People didn't even know the name well. They'd heard it on the radio, heard that the microbe was in the river water. (Male farmer)

Interpretation of first cases (skepticism about river contamination)	Some people said, "I'll see if this water can kill me"…They'd tell you that they've been living here since birth and the water was what their grandfather used for lots of things. (Male community representative)
Current knowledge (transmission)	Here's how a person can get cholera from someone who has it. There are people living in a house, one has it and the [man] doesn't. She's interacting with him, she's not washing her hands. Even if she goes to the hospital with the person, the doctors say that when she gets back home, she should change her clothes before going inside. But when she gets there, instead of changing her clothes, she…goes into the kitchen or holds a baby. The child who didn't have it, now could get cholera too. (Female participant)
	The first and greatest thing is that cholera is friend with dirtiness…The first way that a person gets attacked, like the thing that is less protected – the cholera advice that people neglect is handwashing. (Male community representative).
	There's a [book] that says, 'People who are clean don't get sick easily.' I suppose that the people who got cholera later are those who didn't practice good personal hygiene. (Male tailor)

Current knowledge (cholera prevention)	Wash the legumes very well, cook them thoroughly before eating them. (Male farmer)
	Avoid open defecation (Female community representative)
Cholera treatment (first cases)	[O]ne night, two people were attacked by the disease…We thought it was just same old diarrhea. So, we made our little remedies for diarrhea. Not too long after, one of them died. As soon as we heard that, we rushed the other to the hospital. (Male farmer)
Cholera treatment (over time)	We have come to understand that as soon as we get the disease, we have to go see a doctor, take the person to the hospital. (Female homemaker)

Table S4. Supplementary data on community vulnerabilities.

**Theme: Community vulnerabilities and perceived risk of cholera**	
Sub-theme	Statement
Community vulnerabilities (occupational risk)	When you walk by the embankments of the rice fields, you're working and you see people squatting…you see the feces on the ground…This person's in the field, he doesn't have water to wash his hands, nothing, [then] proceeds to eat with his hands just like that after defecating. (Male farmer)
Community vulnerabilities (flooding and limited access to basic services)	Even coming here [to a health facility], the person dies on the way because the local road is bad …While still on the road, [or] when the person gets here, doesn't even go through 2 bags of *sewòm* [oral rehydration solution] and dies. (Female homemaker)
	When someone had [cholera], you had to find a motorcycle. Sometimes, you had to make a stretcher, find a way to cross the [flood] water…The person either comes to [the nearest health center] or must take a boat and cross over to Larue to get care. Those who had the means as well - like parents in Saint-Marc would come by car and take them [there]. (Male community representative)

Evaluation of emergency response	This is what they mostly did when the disease was ravaging the country – especially since a lot of people were campaigning for election – they only gave us some water… and a few sachets of *sèwom*. Very poorly, to help us. (Female participant)
	People in the health sector did a noble job. [They] provided a telephone number to call for ambulance services. (Male community representative)

Table S5. Supplementary data on prevention practices.

**Theme: Cholera prevention practices and barriers**	
Sub-theme	Statement
Behavior changes: Yes, improved practices	They've changed, they've changed. Because people used to just…go into the river, fill their buckets and get out. But they avoid it now…People protect themselves a little…When they draw water, they put the water down, put Aquatabs in it to bathe, to use, for children just as adults. (Female farmer)
	Till now, we don't drink the water the same way we used to. […] We don't drink it untreated. We are always cautious. That's why people don't die as much as they used to. (Male participant)
Behavior changes: Little to no improvements	In my locality, the feces are on the ground [and] it doesn't take me two minutes to get to the river. There is a lot of mobilization, telling people to not defecate in the open, but there is no other place. (Female farmer)
Reasons for improved practices	I have four children who had cholera, but I didn't because I always use Aquatabs…Up till now, I still haven't had it. (Female participant)

	Because everyone still has to use the river water, even if you didn't have money, with the way the disease was ravaging people, you had to find a way to send [someone] out to buy Aquatabs. (Female homemaker)
Reasons for inconsistent or unimproved practices	In my locality, it's a miserable situation. I have counted – only a few compounds have toilets in them. (Male farmer)
	You could build an earthen latrine, you know, because you don't have the means, no money to build one with cement. [But] after a month, the flood comes and destroys it. (Male farmer)
